# Preliminary Results on Heparin-Modified Double-Layered PCL and PLA-Based Scaffolds for Tissue Engineering of Small Blood Vessels

**DOI:** 10.3390/jfb13010011

**Published:** 2022-01-27

**Authors:** Patrycja Domalik-Pyzik, Anna Morawska-Chochół

**Affiliations:** Department of Biomaterials and Composites, Faculty of Materials Science and Ceramics, AGH University of Science and Technology, 30-059 Kraków, Poland; morawska@agh.edu.pl

**Keywords:** vascular scaffold, polylactide, polycaprolactone, heparin release, blood vessels tissue engineering

## Abstract

Low-diameter blood vessels are challenging to replace with more traditional synthetic vascular grafts. Therefore, the obvious choice is to try to regenerate small veins and arteries through tissue-engineering approaches. However, the layered structure of native vessels and blood compatibility issues make this a very challenging task. The aim of this study is to create double-layered tubular scaffolds with enhanced anticoagulant properties for the tissue engineering of small blood vessels. The scaffolds were made of a polycaprolactone-based porous outer layer and a polylactide-based electrospun inner layer modified with heparin. The combination of thermally induced phase separation and electrospinning resulted in asymmetric scaffolds with improved mechanical properties. The release assay confirmed that heparin is released from the scaffolds. Additionally, anticoagulant activity was shown through APTT (activated partial thromboplastin time) assay. Interestingly, the endothelial cell culture test revealed that after 14 days of culture, HAECs (human aortic endothelial cell lines) tended to organize in chain-like structures, typical for early stages of vascular formation. In the longer culture, HAEC viability was higher for the heparin-modified scaffolds. The proposed scaffold design and composition have great potential for application in tissue engineering of small blood vessels.

## 1. Introduction

The human organism relies on the proper functioning of the complex network of blood vessels: different types of veins, arteries, and capillaries. Their aim is to transport blood between the heart and other organs, delivering oxygen and nutrients, as well as removing metabolites. This crucial role may be adversely affected by many diseases and conditions that have a direct negative impact on the structure and functions of the vessels. In general, cardiovascular diseases are responsible for the majority of deaths in Europe and are also the most common cause of premature mortality in males [1,2].

The extreme importance of appropriate blood supply means that malfunctioning blood vessels need to be replaced. For over 60 years, synthetic vascular prostheses fabricated from various woven and nonwoven polymeric materials have been used in place of larger blood vessels. They are mainly made of poly(tetrafluoroethylene) (PTFE) known as Teflon^®^ or Gore-Tex^®^, and poly(ethylene terephthalate) (PET) known as Terylene^®^ or Dacron^®^ [3]. Their surface can be additionally modified with natural coatings, e.g., albumin, type I collagen, or fibrin to improve their biocompatibility [4,5]. Increased endothelialization of the lumen of a synthetic graft can be also achieved by polydopamine or gelatin modification [6]. Of course, anti-thrombogenic medications are administered to patients with such prostheses to increase the safety and durability of the implant.

Unfortunately, synthetic polymer grafts cannot be used for vessels smaller than 6 mm in diameter, as they can cause rapid clot formation and intima hyperplasia. There is also an increased risk of bacterial infection and chronic inflammatory response. From a mechanical point of view, compliance mismatch between the graft and the vessels (0.5–1.5%/100 mmHg and 5–15%/100 mmHg, respectively) is considered a major drawback, as it can lead to implant rejection. Finally, their inability to grow and rebuild limits their application in the case of pediatric patients. Bearing this in mind, a tissue-engineering approach aiming at recreating a natural blood vessel instead of replacing it with a synthetic imitation seems promising [7].

The original concept of Weinberg and Bell [8] which started the research on tissue-engineered blood vessels was then followed by many other researchers working in the field of vascular tissue engineering. Various approaches using a combination of scaffolds, cells, and bioactive molecules were tested, as well as scaffold-free methods, such as cell-sheet-based and cell-ring-based [9,10,11,12,13,14].

Scaffolds for tissue engineering of small blood vessels need to be biocompatible and hemocompatible, biodegradable, characterized by mechanical properties close to that of a vessel, and with an inner surface that favors endothelium formation. The inside part of the scaffold will be in contact with blood, and therefore should be athrombogenic in order to prevent clot formation. It is especially important for small-diameter scaffolds, due to increased graft surface area to blood volume and slower blood flow. They both result in larger activation of blood elements, and at the same time, their prolonged exposure to contact with the lumen of the scaffold.

Anticoagulant activity of a vascular scaffold can be obtained through the incorporation of heparin or heparin-like modifications of polymeric matrices [14,15,16]. Heparin is considered a “polypharmaceutical”; this natural polysaccharide, besides preventing clot formation, is also able to promote anti-inflammatory behavior, regulate angiogenesis, and presents anti-cancer and antiviral activity [15,17,18].

The aim of our work is to fabricate and preliminarily characterize bioinspired, layered scaffolds with enhanced anticoagulant activity for tissue engineering of small blood vessels. Tubular scaffolds were prepared using poly(L-lactide) and polycaprolactone by combining electrospinning and thermally induced phase separation. Those two methods were coupled to create a gradient and hierarchic microstructure of the scaffold with varying properties of the inner and outer layer dedicated for different types of cells. Heparin, as a potent anticoagulant agent, was introduced into the polymer matrix.

## 2. Materials and Methods

### 2.1. Tubular Scaffolds Fabrication

**Figure 1:**
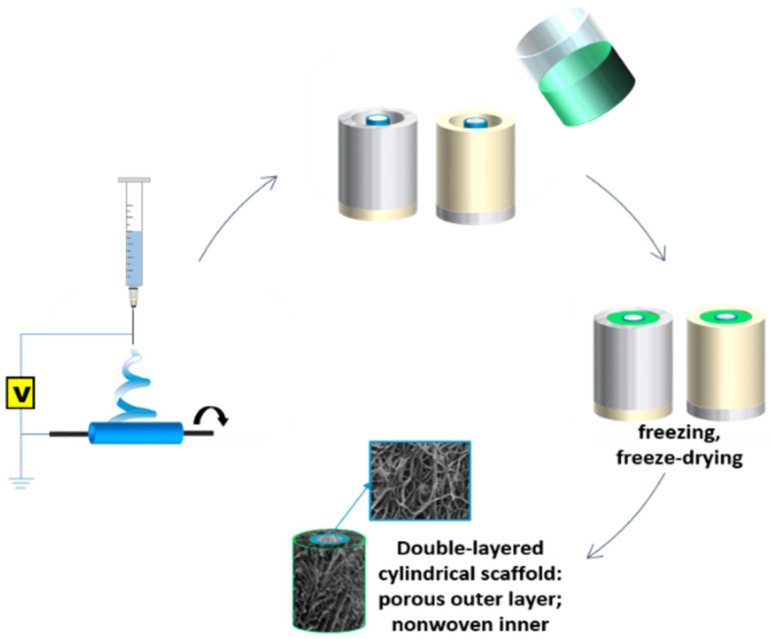
Scheme of the fabrication method of double-layered cylindrical scaffolds using electrospinning and thermally induced phase separation.

Cylindrical, double-layered scaffolds with an inner diameter of 5 mm were fabricated by a combination of electrospinning (ES) and thermally induced phase separation (TIPS), as schematically shown in Figure 1. The inner layer of the proposed scaffold consisted of an electrospun poly(L-lactide) (PLA; IngeoTM 3051D, Nature Works LCC, Minnetonka, MN, USA)-based nonwoven tube. A measurement of 5 wt% PLA solution in chloroform:methanol 3:1 was electrospun (TIC 1092012, Bielsko-Biala, Poland) onto a cylindrical (d = 5 mm) collector with following parameters: applied voltage 15 kV, collector revolutions 330 rpm, the distance between the tip and the collector 20 cm, needle diameter 0.7 cm. Additionally, 5PLA_ES__0.5Hep nonwoven tubes modified with heparin (375095 Heparin, Sodium Salt, Porcine Intestinal Mucosa; Merck Millipore, Darmstadt, Germany, 100KU) were obtained by adding heparin dispersion in the mixed solvents to the PLA spinning solution (final heparin concentration 0.5% *w*/*w* per dry polymer mass), mixing on a magnetic stirrer for 24 h and then sonicating for 5 min. The obtained nonwoven 5PLA_ES_ and 5PLA_ES__0.5Hep tubes were then transferred to cylindrical polytetrafluoroethylene/stainless steel molds where the porous outer layer was produced using the TIPS method. Briefly, a polycaprolactone (PCL; Mw = 80 000, Merck KGaA, Darmstadt, Germany; previously Sigma-Aldrich) solution (2.5 wt% in 99.5–99.9% acetic acid, analytical grade) was poured into the molds containing the electrospun tubes, placed in a −80 °C refrigerator for 6 h and freeze-dried for 96 h. The samples were stored in a desiccator prior to characterization. All of the solvents and phosphate-buffered saline (PBS) reagents were obtained from Avantor Performance Materials Poland S.A, Gliwice, Poland.

### 2.2. Characterization Methods

#### 2.2.1. Microstructure Analysis

The microstructure of the scaffolds was characterized with the scanning electron microscope (SEM; Nova NanoSEM 200, FEI, Eindhoven, The Netherlands). Prior to the observation, the samples were attached to holders with conductive tape and coated with a thin layer of carbon (under vacuum). SEM analysis was carried out with an accelerating voltage of 10.0 or 18.0 kV in the Laboratory of Scanning Electron Microscopy and Microanalysis (Department of Silicate Chemistry and Macromolecular Compounds, Faculty of Materials Science and Ceramics, AGH University of Science and Technology, Krakow, Poland). Quantitative analysis of the diameter of the fibers was performed using ImageJ [19]. One hundred fibers were measured to prepare histograms.

#### 2.2.2. Mechanical Properties

Mechanical properties were evaluated using the universal testing machine Zwick 1435 (ZwickRoell GmbH & Co. KG, Ulm, Germany). Rectangular samples with a dimension of 3 × 35 mm were tested at a crosshead speed of 50 mm/min. Average values of the tensile strength (R_m_), Young modulus (E), and strain at maximum load (ε F_max_) were calculated from at least six independent measurements. The results were presented as mean ± standard deviation for six samples of each type. The results were found statistically significant if *p* < 0.05 according to the *t*-test.

#### 2.2.3. Direct Heparin Release Assay

Two types of heparin release assays were performed. In the direct test, 5PLA_ES__0.5Hep and 5PLA_ES__0.5Hep/2.5PCL_TIPS_ samples were incubated in a phosphate-buffered saline at 37 °C. At fixed time points, 0.6 mL of the solution was sampled and supplemented with fresh PBS. An aliquot of the supernatant was then reacted with 8 × 10^−5^ M azure A chloride solution (certified by the Biological Stain Commission, Dye content 70 %, Merck KGaA, Darmstadt, Germany; previously Sigma-Aldrich) and the heparin release was determined spectrophotometrically (Cecil Instruments BioQuest CE 2502, Cambridge, UK) at 510 nm and 630 nm, using colorimetric method. The assay was performed in triplicate.

#### 2.2.4. Indirect Heparin Release Assay

The indirect heparin release test was conducted by determining activated partial thromboplastin time (APTT). The assay was performed for 5PLA_ES__0.5Hep, 5PLA_ES__0.5Hep/5PCL_TIPS,_ and corresponding samples without heparin as a control. The tested samples were incubated with a known amount of human citrate plasma at 37 °C. APTT was assessed after 1, 3, 4, and 5 h of incubation.

#### 2.2.5. Cytotoxicity Assay

The human aortic endothelial cell line (HAEC, Gibco^®^, ThermoFisher Scientific, Waltham, MA, USA) was used for direct cytotoxicity studies. The cell culture was carried out in endothelial cell growth basal medium-2 (EBM^TM^-2, Lonza) supplemented with fetal bovine serum (FBS), vascular endothelial growth factor (VEGF), insulin-like growth factor 1 (IGF-1), recombinant human epidermal (rhEGF), and fibroblast (rhFGF) growth factors, ascorbic acid, hydrocortisone, and gentamicin sulphate, under standard conditions, i.e., 37 °C, 5.0% CO_2_. Round, 11 mm-diameter samples were sterilized under UV lamp (30 min per side). The sterile samples were transferred into a 48-well plate, then 7 × 10^3^ cell/mL HAEC suspension was added to each well. Cell morphology was evaluated with an optical fluorescence microscope (Olympus CX41, Tokyo, Japan). The cells were stained with 20 µL of acridine orange prior to observation. HAEC viability was assessed using the colorimetric method with CellTiter 96^®^ test (Promega, Madison, WI, USA). Briefly, 40 µL of the reagent was added to each well and incubated at 37 °C, 5.0% CO_2_ for 90 min. Next, 100 µL of the medium with the reagent was transferred to a 96-well plate and the absorbance was measured spectrophotometrically (POLARstar OMEGA, BMG Labtech, Ortenberg, Germany) at 490 nm. The samples were tested in triplicate, tissue-culture polystyrene (TCPS) was used as a control. The results were presented as mean ± standard error of the mean. Statistical analysis was carried out by one-way-ANOVA with post hoc LSD Fisher test (significance level 0.05).

## 3. Results and Discussion

### 3.1. Microstructure and Mechanical Properties

**Figure 2:**
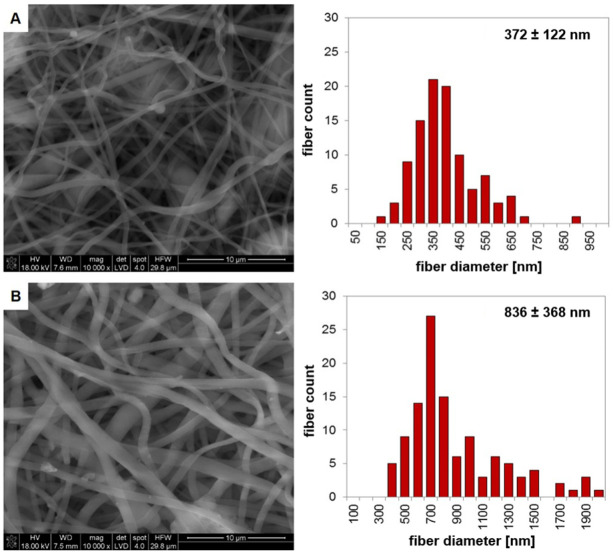
SEM images of the electrospun tubes with histograms of fiber diameter: (**A**) 5PLA_ES_, (**B**) 5PLA_ES__0.5Hep.

Nonwoven tubes based on PLA or heparin-modified PLA were produced using the electrospinning technique. As shown in Figure 2, resulting fibers were beadless and randomly oriented in both cases, but their morphology differed. The addition of the anticoagulant agent (Hep) to the spinning solution resulted in an increase in the mean fiber diameter (more than twice) and an increase in fiber heterogeneity (fiber diameter in the range of ~400–2000 nm) in the case of PLA_ES__Hep tubes. Characteristics of the nonwovens obtained through the electrospinning process were affected by the process parameters, including the spinning solution properties [20]. It can be suspected that heparin altered the viscosity and surface-free energy of the spinning solution. Similar results were observed by A.S. Richard and R.S. Verma [21]. Their results showed an increased PLA-based fiber diameter after the introduction of curcumin. However, it should be noted that the reverse effect was also observed, since the type and properties of the additive affect the final result. A. Magiera et al. showed a decrease in fiber diameter as a result of the addition of carbon nanotubes (CNT) to the PLA solution. The diameter of the PLA/CNT fibers was significantly lower due to the fact that CNT increased the electrical conductivity of the polymer solution and favored jet splitting in the electrical field [22].

The designed small-diameter layered scaffolds should be eventually replaced by the cells. Therefore, contrary to permanent grafts, they need to mimic the mechanical performance of blood vessels only for a limited time, in direct link with the rate of new tissue formation. Of course, in the beginning, their mechanical properties should be sufficient. It is then important to evaluate the mechanical characteristics of the proposed scaffolds. In their recent review, D.B. Camasao and D. Mantovani [23] stated that although the applicability of conventional mechanical tests of the blood vessels’ substitute materials for a full mechanical characterization in relation to the intended application is limited, they still provide important information from the material-science perspective. It is then obligatory to perform a thorough mechanical characterization of the scaffolds in various conditions, including pressure-based tests that enable evaluation of burst pressure and compliance values. Here, the initial mechanical property assessment was performed through the tensile test.

**Figure 3:**
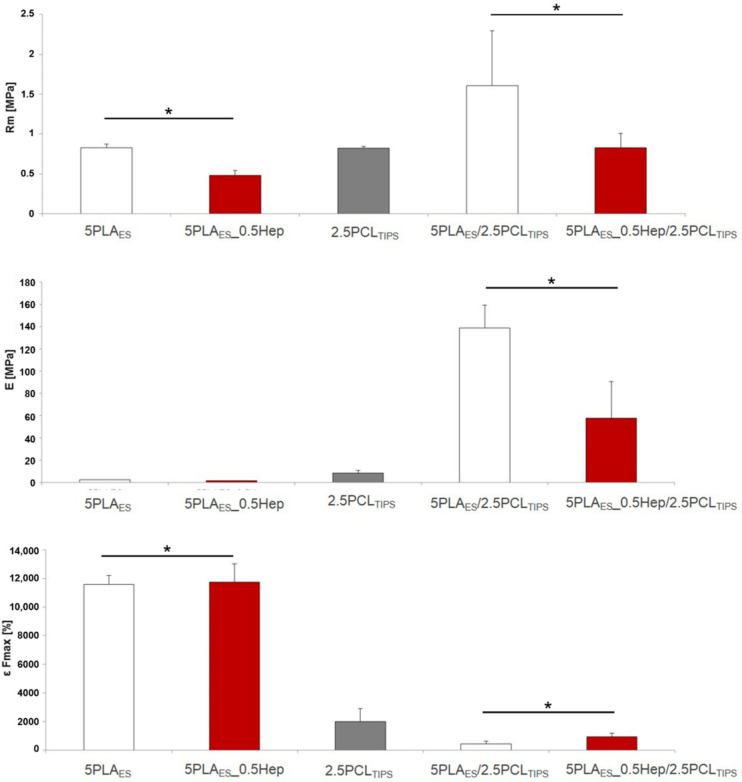
Mechanical properties of the samples: Rm—tensile strength; E—Young modulus; ε Fmax—stress at maximum load. Results are shown as mean values ± standard deviation (SD); * *p* < 0.05.

**Figure 4:**
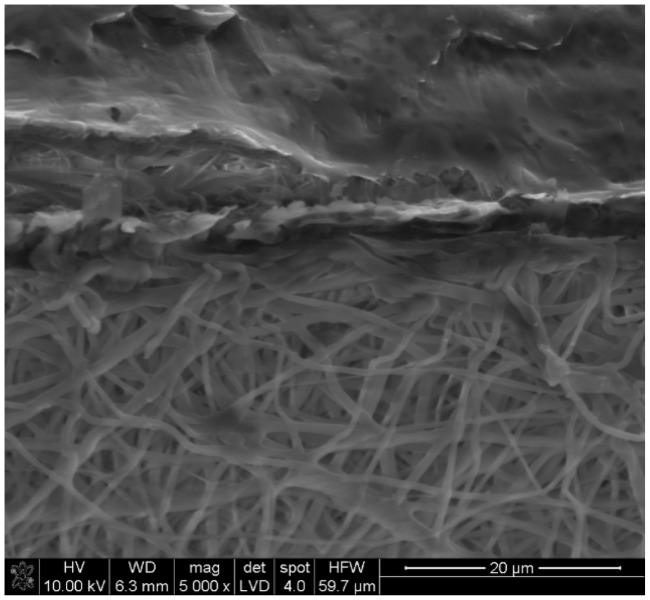
SEM image of the cross section of the 5PLA_ES_/2.5PCL_TIPS_ sample: lower part—PLA nonwoven; upper part—fragment of PCL outer layer.

Analysis of the mechanical properties (Figure 3) confirmed that the PLA nonwovens showed high strain values and a low Young modulus. The high strain of the nonwovens was related to the straightening-up of the fibers during the uniaxial tensile test. The layered scaffolds (porous outer and nonwoven inner) had higher tensile strength and Young modulus, together with lower strain values, as compared to both nonwovens (PLA_ES_ and PLA_ES__0.5Hep) alone. This was probably due to the partial impregnation of highly elastic, PLA-based electrospun fibers with the PCL solution and the formation of an intermediate layer (Figure 4). It can be also noticed that the scaffolds with heparin had lower tensile strength than those based on pure PLA. As already discussed, PLA_Hep nonwovens were also characterized by higher mean fiber diameter values. Previous studies showed that larger fibers may have more defects and decreased molecular-level orientation within the fibers in comparison to their thinner equivalents. Usually, with the increased diameter of electrospun fibers, mechanical properties decrease [24].

H. Mi et al. [25] tested the mechanical behavior of single, double, and triple-layered scaffolds based on thermoplastic polyurethane (TPU) and poly(propylene carbonate) (PPC) fabricated by combining similar methods of electrospinning and thermally induced phase separation. The results were compared to the values for the porcine coronary artery. The test conditions differed, but they also confirmed that layered structures are a better match to native vessels in terms of mechanical behavior.

### 3.2. Heparin-Modified Scaffolds

In order to biologically activate the inner surface of the scaffold and demonstrate the possibility of manufacturing systems for carriers of bioactive substances, heparin was introduced into the spinning solution. As mentioned previously, Hep is known for its anticoagulant properties and ability to hinder smooth muscle-cell proliferation.

**Figure 5:**
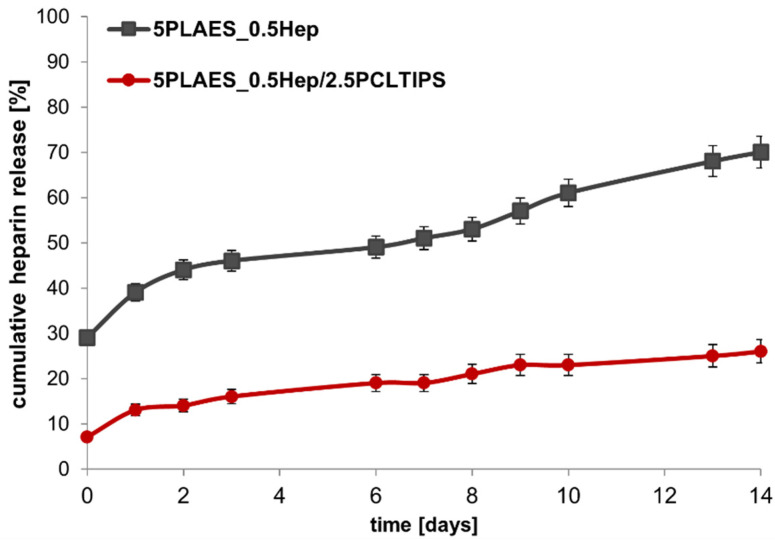
Cumulative heparin release from 5PLA_ES_ nonwoven tube and 5PLA_ES_/2.5PCL_TIPS_ scaffold.

The study was conducted for two types of scaffolds: 5PLA_ES__0.5Hep nonwoven and 5PLA_ES__0.5Hep/2.5PCL_TIPS_ layered scaffold. Differences in the amount of released heparin were observed (Figure 5). A higher release rate was found for the polylactide-based nonwoven. The release mechanism was a diffusion in the water environment (PBS, 37 °C). In the case of the porous, layered scaffold, the addition of the PCL layer, hampered the diffusion possibility by reducing the contact of the surface of PLA-Hep fibers with the medium. For both systems, the release was continued for the duration of the analyzed time (14 days).

The gradual release of heparin over time has been proven to have a positive effect on long-term antithrombotic activity. S. Bae et al. observed the tested release of heparin from PLGA/PEO yarns. It was possible to adjust the release rate through electrostatic interactions, and the best was 65% at 20 days of incubation [26]. In our studies, we obtained the layered scaffolds with long-term heparin release thanks to the introduction of heparin directly into the electrospun fibers and by their combination with a porous PCL layer.

Anticoagulant properties of the designed materials, particularly those modified with heparin, were assessed by identifying the activated partial thromboplastin time. APTT is a typical parameter of blood coagulation that gives information about, for example, activity of blood-plasma-related coagulation factors and the transformation of fibrinogen to fibrin.

**Figure 6:**
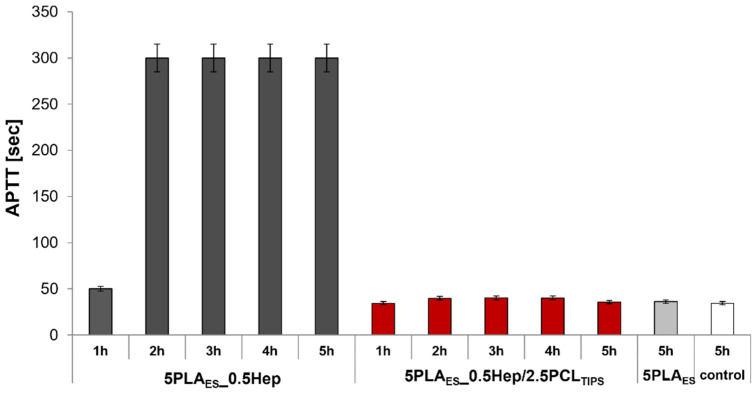
Activated partial thromboplastin time (APTT) determined for 5PLA_ES__0.5Hep nonwoven and layered 5PLA_ES__0.5Hep/2.5PCL_TIPS_ scaffold.

APTT in the range of 25.3–33.8 sec was accepted as normal. The test results showed that the reference material (unmodified PLA_ES_ nonwoven) has APTT similar or slightly above the accepted norm (Figure 6). In the case of heparin-containing nonwoven, the release of heparin was confirmed indirectly. After only 1 h, the APTT for PLA_ES__0.5Hep nonwoven was higher than the reference range. After another hour, until the end of the test, APTT reached the values exceeding the measurement capability of the equipment (>300 s). This confirmed strong anticoagulant properties of the nonwoven modified with Hep. As discussed earlier, the addition of a porous PCL layer hindered heparin release; the APTT values for 5PLA_ES__0.5Hep/2.5PCL_TIPS_ were lower, but still within the accepted range. Thus, it can be concluded that despite the lack of significant anticoagulant properties, the layered scaffolds did not induce coagulation. This is considered to be an advantage in view of their possible application as scaffolds for the tissue engineering of small blood vessels.

### 3.3. In Vitro Assessment of Biological Properties

Previously, we showed that polymeric films made of polylactide and polycaprolactone are able to enhance the angiogenic potential of human umbilical-cord-derived mesenchymal stem cells [27]. Here, three-dimensional scaffolds were produced using the same polymers.

**Figure 7:**
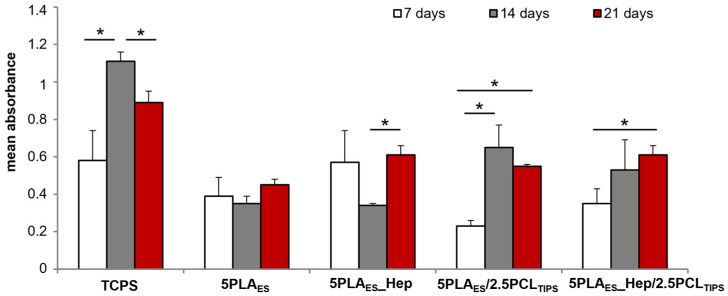
Viability of human aortic endothelial cells (HAEC) after 7, 14, and 21 days of culture on the surface of nonwoven and layered scaffolds. Tissue-culture polystyrene (TCPS) was used as a positive control. Results are shown as mean values ± standard deviation (SD); * *p* < 0.05.

In order to preliminarily assess the biological performance of the designed scaffolds under in vitro conditions, cytotoxicity assay was performed in direct contact with the human aortic endothelial cells (HAEC). The cells’ viability was the highest in the case of a reference surface, i.e., TCPS, and on the heparin-modified nonwoven (5PLA_ES__0.5Hep) (Figure 7). Values for PLA-only nonwoven and layered 5PLA_ES__0.5Hep/2.5PCL_TIPS_ scaffold were only slightly lower. However, in the case of the layered scaffold without heparin, the number of live cells after 7 days of culture was nearly three times lower than for the control. In the longer period (the culture was maintained for 21 days), the lowest viability was seen for PLA_ES_. The viability of the cells cultured on the surface of the remaining scaffolds was similar or around 25% lower than in the case of TCPS. No cytotoxicity was observed. It is also noteworthy that the viability of the cells cultured on layered scaffolds increased with the culture time.

**Figure 8:**
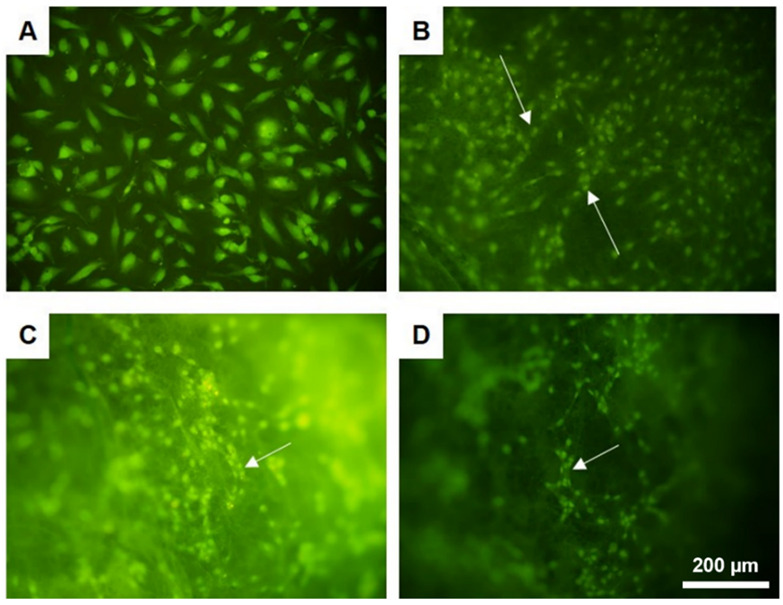
Morphology of human aortic endothelial cells (HAEC) after 14 days of culture on the surface of (**A**) TCPS, (**B**) 5PLA_ES_/2.5PCL_TIPS_, and (**C**,**D**) 5PLA_ES__0.5Hep/2.5PCL_TIPS_ (staining: acridine orange; scale bar is 200 µm).

Additionally, the cells were visualized under a fluoroscopic microscope (Figure 8). It should be noted that imaging of cells cultured on 3D nonwoven scaffolds is especially difficult. The cells tend to migrate in between the fibers, hindering imaging of individual cells. Moreover, the nonwoven may become ridged, creating folds and hiding the cells. Therefore, distinct morphologies of singular cells were not as clear as in the case of the TCPS surface. However, it was visible that after 14 days of culture, the cells tended to organize in chain-like structures. That is particularly interesting, since similar behaviour occurs at the early stages of vascular formation.

## 4. Conclusions

In this study, double-layered scaffolds made of PCL-based porous outer layer and PLA-electrospun inner layer modified with heparin were proposed for the tissue engineering of small blood vessels (below 6 mm in diameter). The combination of thermally induced phase separation and electrospinning resulted in asymmetric scaffolds with improved mechanical properties. Both direct and indirect tests confirmed that heparin is released from the scaffolds. Additionally, anticoagulant activity was shown through APTT assay. Interestingly, the endothelial cell culture test revealed that after 14 days of culture, HAEC tended to organize in chain-like structures, typical for early stages of vascular formation. In the longer culture, HAEC viability was higher for the heparin-modified scaffolds. The proposed scaffold design and composition have the potential for application in the tissue engineering of small blood vessels.

## Data Availability

The data presented in this study are available on request from the corresponding author.
